# Sedative and Anxiolytic-Like Actions of Ethanol Extract of Leaves of* Glinus oppositifolius* (Linn.) Aug. DC.

**DOI:** 10.1155/2016/8541017

**Published:** 2016-06-16

**Authors:** Md. Moniruzzaman, Partha Sharoti Bhattacharjee, Moushumi Rahman Pretty, Md. Sarwar Hossain

**Affiliations:** Department of Pharmacy, Stamford University Bangladesh, 51 Siddeswari Road, Dhaka 1217, Bangladesh

## Abstract

*Glinus oppositifolius *is a small herb, widely used in the traditional medicine of Bangladesh in treatment of a variety of diseases and disorders such as insomnia, pain, inflammation, jaundice, and fever. The present study evaluated the sedative and anxiolytic potentials of the ethanol extract of leaves of* G. oppositifolius* (EEGO) in different behavioral models in mice. The sedative activity of EEGO was investigated using hole cross, open field, rotarod, and thiopental sodium- (TS-) induced sleeping time determination tests, where the elevated plus maze (EPM) and light-dark box (LDB) exploration tests were employed to justify the anxiolytic potentials in mice at the doses of 50, 100, and 200 mg/kg. The results demonstrated that EEGO significantly inhibited the exploratory behavior of the animals both in hole cross and in open field tests in a dose-dependent manner. It also decreased motor coordination and modified TS-mediated hypnosis in mice. In addition, EEGO showed anxiolytic potential by increasing the number and time of entries in the open arm of EPM, which is further strengthened by increase in total time spent in the light part of LDB. Therefore, this study suggests the sedative and anxiolytic properties of the leaves of* G. oppositifolius *and supports the traditional use of this plant in treatment of different psychiatric disorders including insomnia.

## 1. Introduction

Anxiety and insomnia are the most prevalent physiological and psychological states characterized by cognitive, emotional, and behavioral components affecting one-eighth of the world population [1]. Nowadays, several sedative drugs (e.g., diazepam, which is chosen as reference standard in this study) come with sanative potential to manage sleeping disorders, which also could reduce anxiety [2, 3]. However, in addition to their beneficial properties, these currently available sedative and anxiolytic therapies possess serious adverse and side effects. Therefore, newer, more efficacious and better-tolerated treatments including alternative/complementary medicines would be a welcome addition in the therapeutic repertoire of insomnia and anxiety management.

*Glinus oppositifolius* (family: Molluginaceae) is a very common herb in Bangladesh, locally known as “gima shak.” This plant is extensively used by the local people as a vegetable and the traditional healers as an essential ingredient in treatment of a wide range of diseases and disorders including pain, inflammation, jaundice, diarrhea, boils, and malaria [4]. This herb is also useful for its diuretic, CNS depressant, anthelmintic, and antiviral properties in the folk medicine of this country [5]. In recent years, several studies have been conducted to identify the phytochemical constituents of* G. oppositifolius* and a number of phytochemicals including L-(−)-(*N*-*trans*-cinnamoyl)-arginine, kaempferol 3-*O-*galactopyranoside, isorhamnetin 3-*O*-*β*-D-xylopyranosyl-(1→2)-*β*-D-galactopyranoside, and L-phenylalanine have been isolated from different parts of this plant [6]. Further studies have revealed that* G. oppositifolius* also contains spergulagenin derivatives [4], oppositifolone [7], and a bioactive pectic polysaccharide having immunomodulatory properties [8]. According to the aforementioned medicinal properties and the presence of different bioactive phytochemicals, researchers tried to validate its traditional uses against different diseases and disorders. Their findings revealed that* G. oppositifolius *is effective as an antioxidant, hypoglycemic, anti-inflammatory [4], antidiarrheal, and anthelmintic [5] agent. However, till now there is no scientific report revealing its actions on the central nervous system. This influenced us to design and conduct the present study to evaluate the impact of ethanol extract of leaves of* G. oppositifolius* in different behavioral models in mice.

## 2. Materials and Methods

### 2.1. Plant Collection and Extraction

*Glinus oppositifolius* leaves were collected from Khilgaon, Dhaka, during the spring of 2013. The collected samples were then identified by Mr. Sardar Nasir Uddin, Senior Scientific Officer, Bangladesh National Herbarium (Mirpur, Dhaka, Bangladesh). A voucher specimen has been deposited with a number DACB: 38355 for further references. The powdered dried leaves (250 g) were macerated with 450 mL of ethanol (100%; Merck, Bremen, Germany) with occasional stirring at room temperature for three days. Then the filtrate was collected and completely dried using a rotary evaporator. Finally 11.83 g extract (yield 4.73%) was obtained which was further used in the entire set of studies.

### 2.2. Animals

Adult Swiss albino mice (20–25 g) were purchased from the Animal Resources Branch of the International Center for Diarrheal Disease Research, Bangladesh (icddr,b), and housed in standard laboratory conditions (relative humidity 55–60%; room temperature 25 ± 2°C; 12 h light/dark cycle) with standard diet (icddr,b formulated) and water* ad libitum*. Animals were acclimatized with the experimental environment for a period of 14 days prior to the experiments and then treated according to the “Ethical Principles and Guidelines for Scientific Experiments on Animals” (1995) drafted by the Swiss Academy of Medical Sciences and the Swiss Academy of Sciences. All experimental protocols employed in this study were approved by the Institutional Ethics Committee of Stamford University Bangladesh (SUB/IAEC/14.08).

### 2.3. Drugs and Treatments

Mice were divided into five groups containing 5–7 animals each for control, standard, and test samples, for every experiment. Standard drug diazepam (1 mg/kg; i.p.) (Square Pharmaceuticals Ltd., Dhaka, Bangladesh), EEGO (50, 100, and 200 mg/kg; p.o.), or vehicle (DMSO; 0.1 mL/mouse; p.o.; Merck), was administered to the animals, immediately after taking the pretreatment reading in hole cross and open field tests. For the rest of the models, diazepam was administered at 15 min and EEGO or vehicle at 30 min before the experiments. In sleeping time determination test, the sleep inducer thiopental sodium (20 mg/kg) (Square) was administered 15 min posttreatment with diazepam and 30 min of vehicle or EEGO.

### 2.4. Phytochemical Screening

The crude extract of* G. oppositifolius* leaves was qualitatively analyzed to detect the presence of alkaloids, glycosides, carbohydrates, saponins, flavonoids, tannins, glucosides, and reducing sugars following standard procedures [9].

### 2.5. Acute Toxicity Test

Mice were divided into desired groups each containing 5–7 animals. EEGO was administered to the animals orally at the doses of 500, 1000, and 2000 mg/kg. The mice were then allowed to take food and water* ad libitum* and observed for the next 72 h to check any allergic symptoms and mortality induced by EEGO [10].

### 2.6. Sedative Activity Analysis

#### 2.6.1. Hole Cross Test

A cage having a size of 30 × 20 × 14 cm with a fixed partition in the middle having a hole of 3 cm diameter was used in this experiment [11]. Mice were treated with either vehicle or drug or EEGO and allowed to cross the hole from one chamber to another. The animals were then observed for 3 min and the number of passages was recorded before and at 30, 60, 90, and 120 min following the treatments.

#### 2.6.2. Open Field Test

This test is a widely used model for the evaluation of emotional behavior of the animals, especially the rodents. The method was carried out as described by Gupta et al. (1971) [12]. The open field apparatus consisted of a wooden field of half square meter with a series of squares alternatively painted in black and white. It had a wall of 50 cm height and was placed in a dimly lit room. The animals were placed in the middle of the open field to explore freely and the number of squares visited by them was counted for 3 minutes as pretreatment reading. Immediately after taking the reading the animals were treated with vehicle, extract, or diazepam and observed repeatedly at 30, 60, 90, and 120 min after the treatments.

#### 2.6.3. Test for Motor Coordination (Rotarod Test)

The rotarod test was performed according to the procedure described by Dunham and Miya (1957) [13]. This test is effective for the investigation of motor impairment due to pharmacological agents like muscle relaxants or CNS depressants. The apparatus consisted of a horizontal nonslippery plastic rod, rotating at 20 rpm. The animals which can remain in the rotating rod for more than 180 sec were selected for this study. After desired treatments, each mouse was placed on the rod and the falling time of each mouse within 180 sec was recorded as an indication of muscle relaxation.

#### 2.6.4. Thiopental Sodium-Induced Sleeping Time Determination

Thiopental sodium-induced sleeping time test was performed according to the previously described method [14]. Following desired sample or drug administration, the animals were observed for the latent period (time to lose their righting reflex, immediately after thiopental sodium injection) and the duration of sleep (time between the loss and recovery of reflex) induced by thiopental sodium.

### 2.7. Investigations for the Anxiolytic Potential

#### 2.7.1. Elevated Plus Maze Test

The elevated plus maze (EPM) test is a widely used model to investigate anxiolytic effects. The apparatus consists of two open arms (15 × 5 cm^2^) and two closed arms (15 × 5 × 5 cm^3^), extending from a central platform (5 × 5 cm^2^) and raised 50 cm above floor level. Animals were randomly divided into each group and treated with EEGO, vehicle, or diazepam. After the desired time, each animal was placed at the center of the plus maze facing its head to the closed arms and allowed free exploration for 3 min. Then the number of entries and total time spent in open arms were recorded within the indicated time [15, 16].

#### 2.7.2. Light-Dark Box (LDB) Exploration Test

The apparatus is an open-topped rectangular box (46 × 27 × 30 cm^3^), divided into a small (18 × 27 cm^2^) and a large (27 × 27 cm^2^) compartment with a fixed partition containing a small hole of 3 cm diameter in the middle. The small compartment was closed with a lid, painted black, and illuminated with a dim light. On the other hand, a 60 W electric light was used in the large compartment (painted white) to make it brightly illuminated. After 30 min of successful oral gavage of vehicle, EEGO, or diazepam, mice were placed in the middle of the open compartment of LDB. The animals were then observed for 3 min and the time spent in the lighted compartment and the total number of transitions in between the compartments were registered [17].

### 2.8. Statistical Analysis

The results are presented as Mean ± SEM. The statistical analysis was performed using two-way analysis of variance (ANOVA) followed by Bonferroni's* post hoc* test for hole cross and open field tests, where one-way ANOVA followed by Dunnett's* post hoc* test was employed for other experiments performed in this study. All statistical analyses were performed using SPSS software. Besides, the ED_50_ values were calculated using GraphPad Prism and the figures were drawn using SigmaPlot software.

## 3. Results and Discussion

The present study evaluated the effect of an ethanol extract of* G. oppositifolius* on CNS using several behavioral models in mice. Our results demonstrated that, following oral administration, EEGO was able to promote CNS depressant and anxiolytic effects. Moreover, EEGO at the doses of 500–2000 mg/kg did not produce any allergic manifestation or mortality of the animals during 72 h of observation period, which is in line with the wide dietary and therapeutic uses of this plant. This nontoxic profile of the extract also made us capable of choosing the experimental doses in this study.

**Figure 1:**
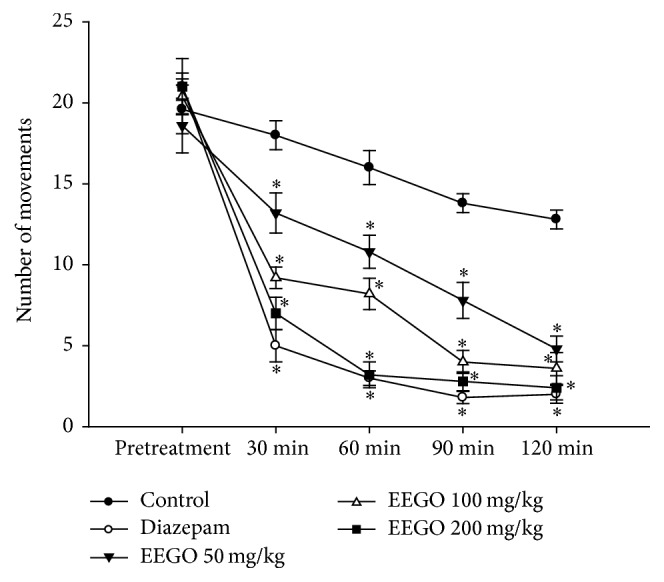
Effect of EEGO on hole cross test in mice. Before and after treatments with diazepam, vehicle, or EEGO the number of holes crossed in the hole cross box was recorded at different time points. Data were presented as Mean ± SEM (*n* = 5–7). ^*∗*^ *p* < 0.01 compared to control.

**Figure 2:**
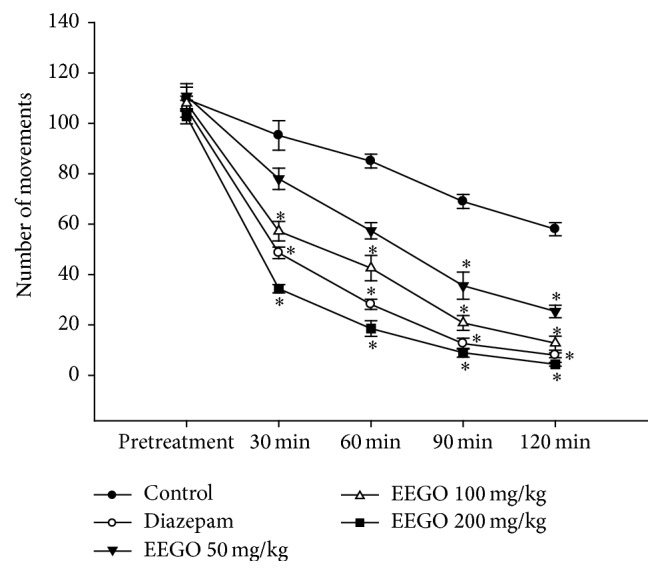
Effect of EEGO on open field test in mice. Before and after treatments with diazepam, vehicle, or EEGO the number of squares crossed in the open field box was recorded at different time points. Data were presented as Mean ± SEM (*n* = 5–7). ^*∗*^ *p* < 0.01 compared to control.

**Figure 3:**
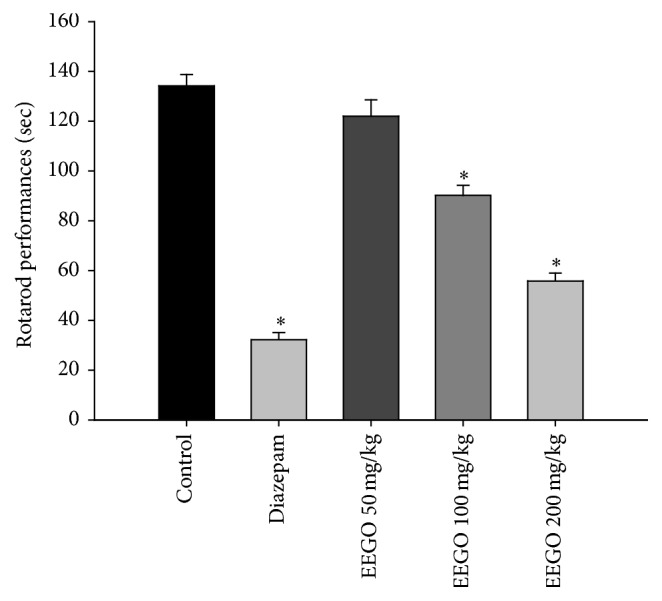
Effect of EEGO on motor coordination of mice. Thirty min after the treatment with EEGO or vehicle and 15 min after diazepam, rotarod performances by the animals were observed for 180 sec. Data were presented as Mean ± SEM (*n* = 5–7). ^*∗*^ *p* < 0.01 compared to control.

The hole cross and open field tests are the most common experimental models used to investigate the exploratory behavior of the animals. It is well established that several drugs like benzodiazepines suppress curiosity of the animals about a new environment resulting in a decrease in their locomotor activity [11, 18]. Likewise, our results demonstrated that EEGO significantly (*p* < 0.01) reduced locomotion of the animals both in hole cross and in open field tests. The suppressive effect was observed from 30 min and continued up to 120 min of EEGO administration (Figures 1 and 2) accounting 81 and 92% of locomotor inhibition at the highest time point of these tests, respectively. Besides, it is well established that the CNS depressant drugs like benzodiazepines cause muscle weakness [19], decreased ambulatory activity, and sedation which negatively affect the rotarod performance of the animals [20, 21]. As depicted in Figure 3, EEGO decreased the falling latency of the animals from rotarod, significantly (*p* < 0.01) with the doses of 100 and 200 mg/kg. The highest motor coordination impairment and ED_50_ were calculated as 58% with 200 mg/kg and 89.66 mg/kg, respectively. In parallel, diazepam at 1 mg/kg dose also produced similar pattern of effects observed with EEGO in all experiments. From these observations, it is conceivable that, like diazepam, EEGO may have the potential to act on CNS which was reflected by its locomotor inhibitory activity as well as impaired motor coordination effects in the animals.

**Figure 4:**
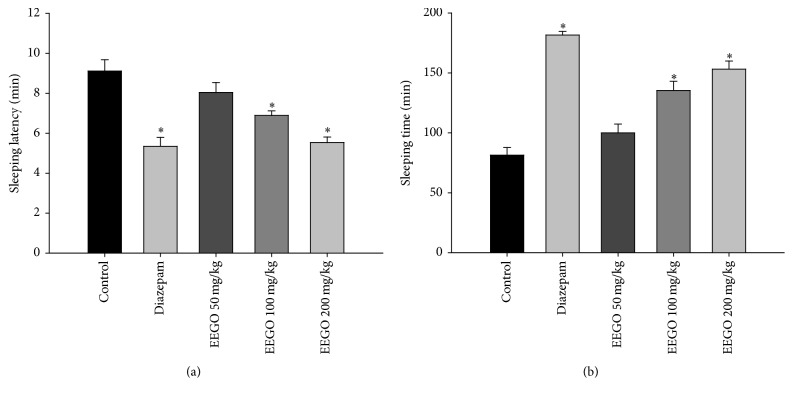
Effect of EEGO on TS-induced hypnosis in mice. Thirty min after the treatment with EEGO or vehicle and 15 min after diazepam, TS was administered intraperitoneally. Then the latency to sleep (a) and total sleeping duration (b) induced by TS were observed. Data were presented as Mean ± SEM (*n* = 5–7). ^*∗*^ *p* < 0.01 compared to control.

Further evidence of the central sedative activity of EEGO is provided by TS-induced sleep enhancing ability of the extract. Substantial scientific reports suggested that the CNS depressant barbiturates like TS bind to the barbiturate binding site of the GABA_A_ receptor, which potentiates GABA-mediated hyperpolarization of the neurons [22]. In our study, the acute oral administration of EEGO significantly (*p* < 0.01) modulated the sleeping behavior of the animals induced by TS. We found that EEGO decreased TS-induced onset of sleeping and increased the sleeping duration in a dose-dependent manner (Figures 4(a) and 4(b)) with the ED_50_ value of 69.73 mg/kg. Therefore, these results strengthened the sedative and muscle relaxation potentials of the extract observed in hole cross, open field, and rotarod tests.

**Figure 5:**
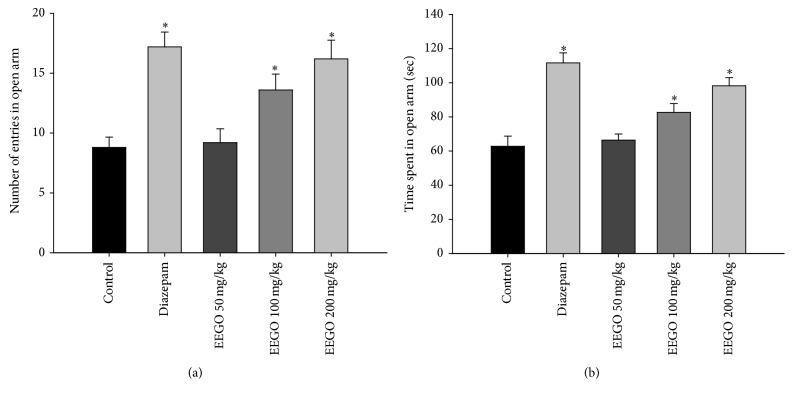
Effect of EEGO on elevated plus maze (EPM) test in mice. Thirty min after the treatment with EEGO or vehicle and 15 min after diazepam, animals were observed for their number of entries (a) and total time spent (b) in the open arms of EPM. Data were presented as Mean ± SEM (*n* = 5–7). ^*∗*^ *p* < 0.01 compared to control.

**Figure 6:**
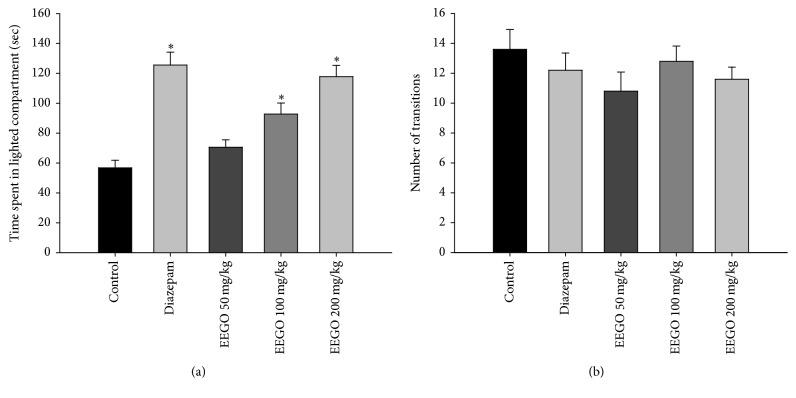
Effect of EEGO on light-dark box (LDB) exploration test in mice. Thirty min after the treatment with EEGO or vehicle and 15 min after diazepam, animals were observed in LDB and the time spent in the lighted part (a) and number of transitions between compartments (b) were recorded. Data were presented as Mean ± SEM (*n* = 5–7). ^*∗*^ *p* < 0.01 compared to control.

**Figure 7:**
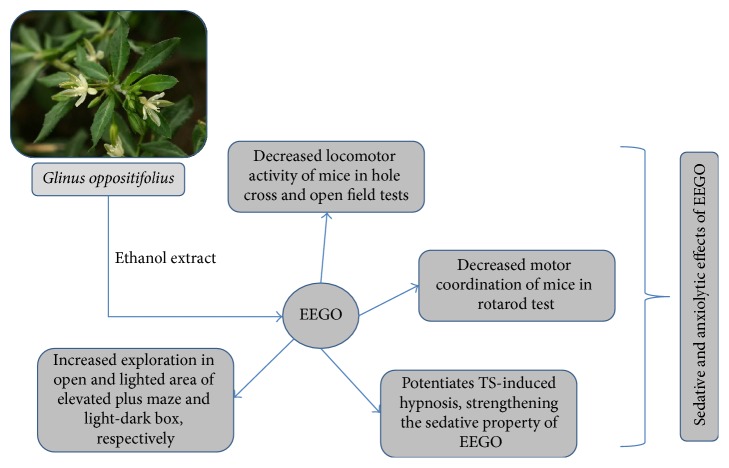
Sedative and anxiolytic activities of EEGO.

The elevated plus maze is a widely used behavioral model in rodents and has been validated to investigate the anxiolytic potential of different pharmacological agents [23]. The open arm activities of the animals in EPM reflect a conflict between the animal's innate behavior to keep itself in a protected area (e.g., closed arms) and motivation to explore in a novel environment, where the anxiolytic agents induce the exploratory activities of the rodents in the open arm [15, 24]. Our results demonstrated that EEGO caused a marked increase in the number of entries as well as the time the animals spent in the open arms of EPM (Figures 5(a) and 5(b)). However, the significant (*p* < 0.01) effect was observed with 100 and 200 mg/kg doses of EEGO and the ED_50_ values were calculated as 88.65 and 92.87 mg/kg for the number of entries and time spent, respectively. In addition, the effect of EEGO was also evaluated using LDB, a popular screening tool in research of anxiolytic or anxiogenic agents [25]. The present study demonstrated that the oral administration of EEGO at 100 and 200 mg/kg doses could significantly (*p* < 0.01) increase the time the animals spent in the lighted area (ED_50_: 82.46 mg/kg) without altering the total number of transitions in between the compartments. As expected, diazepam also exhibited similar patterns of effects of EEGO in these models (Figures 6(a) and 6(b)). Substantial scientific reports demonstrated that, with an anxiolytic drug treatment, animals increased their transitions in between the compartments of LDB. In contrast, there were no changes observed by other researchers when they administered standard anxiolytics to their animals. These discrepancies might be due to the genetic or strain variation of the animals used in their studies. Therefore, it has been concluded that simply the time spent in the lighted area, but not the total number of transitions, is the most useful and consistent parameter to evaluate an anxiolytic action [25]. Therefore, our results and previously published reports suggest that EEGO may possess anxiolytic potentials along with its sedative properties (Figure 7). Although this research has reached its goals, the audience of this paper might have the query regarding the effects of higher doses of EEGO on the above models. Therefore, it is worthy to include few more doses (higher than 200 mg/kg) in the future studies.

Our phytochemical screening of EEGO revealed the presence of alkaloids, carbohydrates, flavonoids, glycosides, saponins, and tannins (Table 1), where saponins are known to show amphetamine antagonism and sedative property and decrease spontaneous motor activity in the experimental animal models [26]. It has also been reported that the presence of alkaloids, glycosides, and flavonoids in plant extract possess sedative and anxiolytic effect through the interaction with GABA_A_ receptors [27–29]. Considering our results and previously published reports, it is possible that the abovementioned chemical components in the extract might contribute at least in part to the observed pharmacological activities. We may, therefore, conceive that the ethanol extract of* G. oppositifolius *contains psychoactive principles that are sedative and anxiolytic in nature.

## 4. Conclusion

Our preliminary pharmacological studies suggest that the ethanol extract of* Glinus oppositifolius* leaves possesses sedative properties, reduces locomotor activity, and causes muscle relaxation in experimental animals, which authorize the possible anxiolytic-like actions of the extract. Therefore, these results provide the scientific validation for the use of this plant in traditional medicine in treatment of various ailments related to CNS disorders. However, further pharmacological studies are required to clearly understand the sedative and anxiolytic actions of EEGO, where our findings could stand as a basis for further progress. In addition, whether these activities were generated by the actions of agonists or partial agonists present in EEGO which could directly act on the receptor(s) responsible and/or interact with other molecular mechanism(s) involved in the observed sedative-anxiolytic effects also needs to be investigated. These bioactivity-guided phytopharmacological works will give us the opportunity to identify pharmaceutical lead(s) with better tolerability and lesser side effects in new drug development.

## Figures and Tables

**Table 1:** Groups of phytochemicals identified in EEGO.

Phytochemicals	Name of the tests	Expected changes	Results
Alkaloids	Mayer's test	Yellowish buff color precipitate	+
	Hager's test	Yellow crystalline precipitate	+
	Wagner's test	Brown or deep brown precipitate	+
	Dragendorff's test	Orange or orange-brown precipitate	+
	Tannic acid test	Buff color precipitate	−

Tannins	Ferric chloride test	Blue-green color	+
	Alkaline reagent test	Yellow to red precipitate	+

Glycosides	General test	Yellow color	+
	Test for glucoside	Production of brick-red precipitation	+

Carbohydrates	Molisch's test	A red or reddish violet ring is formed at the junction of two layers and on shaking a dark purple solution is formed	+
	Barfoed's test (general test for monosaccharides)	Red precipitate	−
	Fehling's test	A red or brick-red precipitate	+
	Test for reducing sugar	A brick-red precipitate	+

Flavonoids	Hydrochloric acid reduction test	Red color	+

Saponins	Frothing test	Formation of stable foam	+
